# State-of-the-Art Treatments for Sarcoidosis

**DOI:** 10.14797/mdcvj.1068

**Published:** 2022-03-14

**Authors:** Ilias C. Papanikolaou, Emmanouil Antonakis, Aggeliki Pandi

**Affiliations:** 1 Corfu General Hospital, Corfu, GR

**Keywords:** sarcoidosis treatment, cardiac sarcoidosis, advanced sarcoidosis, neurosarcoidosis

## Abstract

Sarcoidosis is a heterogeneous disease with various treatment indications. Although it affects mainly the lungs, sarcoidosis can affect every organ, especially when the disease course is chronic and protracted. Regular patient follow-up is recommended for early recognition of active, ongoing organ-specific granulomatous inflammation to avoid progression to irreversible fibrosis. In this review, we elaborate on treatment indications and various anti-sarcoidosis regimens proven useful in clinical trials. We also review specialized treatment of specific disease manifestations, with a focus on cardiac sarcoidosis. We also report on treatment for special conditions such as fatigue and small fiber neuropathy. Treatment for sarcoidosis is an emerging landscape, with new data complementing the existing knowledge.

## Introduction

Sarcoidosis is a multisystem granulomatous disease of yet unknown etiology. It has great predilection for the lungs, but multiorgan involvement is common, especially when the disease course is chronic and protracted. Timely recognition and treatment is important as inflammation may progress to fibrosis, leading to irreversible organ damage. Older as well as recent guidelines for follow-up include identification of symptoms, history and physical examination, pulmonary function testing, and blood tests (complete blood counts, serum creatinine, serum alkaline phosphatase, serum calcium).^1,2^ Depending on symptoms, additional tests may include an eye examination, cardiac investigations, vitamin D assessment, and computed tomography (CT) of the lungs. Cardiac sarcoidosis (CS) occurs in 5% of all sarcoidosis cases, but autopsy and imaging studies reveal clinically silent CS in 25% of cases in the United States.^3^ CS manifests with atrioventricular blocks, arrythmias, heart failure, and sudden cardiac death, accounting for up to 25% of total sarcoidosis deaths; therefore, prompt recognition and treatment is crucial.^4^ CS affects all races, has a mean age presentation of 50 years, and may be isolated without evidence of extracardiac sarcoidosis in 25% of those who have it.^4^ In this review, we address the principles and indications of sarcoidosis treatment, regimens and disease-modifying drugs, and treatment of special conditions.

## Indications for Treatment

Not all sarcoidosis patients need systemic immunomodulatory treatment. This variation occurs because in roughly 30% of cases, the disease may subside by itself without ever being treated.^5^ Also, certain disease manifestations (ie, anterior uveitis, skin disease other than lupus pernio) may be treated only locally. Conversely, a recent British Thoracic Society report states that a significant proportion, up to 30%, of patients develop severe and worsening chronic disease requiring ongoing systemic treatment. Mortality is significant among this subgroup of patients, ranging up to 25%.^6^

The decision to treat with disease-modifying agents requires symptomatic, ongoing, and gradually worsening organ involvement (pulmonary or extrapulmonary) as well as certain asymptomatic but significant extrapulmonary manifestations, namely CS, neurosarcoidosis, posterior uveitis, and hypercalcemia.^1,2^ In other words, treatment is warranted when there is danger of permanent debilitating organ damage or dysfunction with associated morbidity and/or a significantly compromised health-related quality of life on patient-reported outcomes.^6^ Thus, the goal of treatment is to prevent irreversible evolution of active granulomatous inflammation to fibrosis.^7^ Disease activity has to be demonstrated before initiation of treatment because already established organ dysfunction should not be treated; disease activity is not sufficient by itself for treatment initiation when organ dysfunction is absent or mild.

Diagnosis of CS with high probability requires abnormal findings in electrocardiogram and/or Holter monitoring and/or echocardiography with abnormal advanced imaging features at cardiac magnetic resonance (CMR) and/or cardiac fluorodeoxyglucose positron emission tomography (FDG-PET).^8,9^ Isolated CS may be diagnosed clinically in the absence of cardiac and extracardiac histology when positive PET scan combines with clinical features with or without positive CMR.^8,10^

Recent studies have identified key risk factors for increased mortality in pulmonary sarcoidosis, including decreased diffusion capacity of carbon monoxide (DLCO), presence of pulmonary fibrosis in high-resolution CT, and pulmonary hypertension.^11,12^ The ideal regimen and medication doses in sarcoidosis are not always straightforward, because randomized double-blind placebo-controlled trials are generally lacking in sarcoidosis due to disease complexity, heterogeneity, and ethical considerations to withhold established treatments (with corticosteroids). ***Table 1*** summarizes a treatment algorithm of CS.^13,14^ FDG-PET seems to be useful to monitor active myocardial inflammation.^15^ Treatment has not shown to alter the natural course of the disease longitudinally, as granulomas often recur or progress. The most efficient drug against sarcoidosis is undisputedly corticosteroids. The other two categories of drugs with anti-inflammatory effect are antimetabolites and biologic agents.

**Table 1:** Management of cardiac sarcoidosis.


Monitoring	At least yearly or sooner depending on symptoms, disease severity, laboratory parameters (echocardiography, troponin, BNP)

Addressing concomitant cardiovascular risk factors	Coronary artery disease, hypertension

Standard medical treatments of heart failure, diastolic dysfunction	

Managing conduction abnormalities (AV blocks)	Consider pacemaker

Managing ventricular arrythmias, risk for sudden cardiac death	Consider implantable cardioverterdefibrillator

Immunosuppressive treatment*	Administer in functional cardiac abnormalities (arrythmias, myocardiopathy, blocks)® In asymptomatic cases with normal ejection fraction: individualized assessment as per treatment initiation

First-line treatment: corticosteroids	Second-line treatment: methotrexate, leflunomide, azathioprine, mycophenolate mofetil Third-line treatment: infliximab, adalimumab, cyclophosphamide


* Risk factors: Age greater than 50, left ventricular ejection fraction of less than 40%, New York Heart Association functional class 3 or 4, increased left ventricular end-diastolic diameter, late gadolinium enhancement on cardiac MRI, ventricular tachycardia, cardiac inflammation identified by fluorodeoxyglucose positron emission tomography (FDG-PET) scan, echocardiographic evidence of abnormal global longitudinal strain, interventricular septal thinning, elevated troponin or BNP. BNP: brain natriuretic peptide; AV: atrioventricular ®: Monitor myocardial inflammation with FDG-PET

## Treatments

### Corticosteroids

Corticosteroids remain the mainstay first-line treatment of sarcoidosis. They have been proven to improve overall disease control, symptoms, quality of life, and pulmonary radiology and/or delay disease deterioration in patients with symptomatic pulmonary sarcoidosis. These effects are short-term, lasting for a period of 1 to 2 years, and fade after treatment withdrawal.^16,17,18,19,20^ Note that oral corticosteroids may not have an effect on pulmonary physiology as expressed by forced vital capacity (FVC) and diffusing capacity for carbon monoxide. Furthermore, inhaled corticosteroids, commonly used, have proven ineffective in clinical trials.^21^

Studies show that an initiating dose of prednisone at 20 mg daily is not inferior to higher doses in clinical efficacy, control of the disease, physiology, or quality of life parameters.^22,23,24^ In recent studies, these relatively moderate doses are shown to manage sarcoidosis manifested as acute disease or as flares of chronic disease, and they are currently recommended by major societies (British Thoracic Society, European Respiratory Society) as an initiation dose with specific exceptions.^6,14^ These guidelines consider the significant toxicity of treatment with corticosteroids associated with both cumulative dose and duration of treatment. Common side effects including weight gain, diabetes, glaucoma, osteoporosis, and depression have been frequently observed in sarcoidosis patients.^25^

When initiating corticosteroids in a patient with sarcoidosis, close follow-up every 3 months is mandatory. The initial dose is tapered after 3 to 6 weeks, usually by 5 mg, targeting a maintenance dose of 5 to 10 mg of prednisone by approximately 6 months after initiation of treatment. The maintenance dose is required by the majority of patients for a period of 6 to 24 months.

Based on existing literature, several principles apply when considering treating pulmonary sarcoidosis with corticosteroids: (1) acute disease and flares of chronic disease require the same prednisone doses, (2) the chronic maintenance dose should be the lowest possible, and (3) treatment with corticosteroids should be fairly prolonged to allow clearance of the etiologic antigen and adequate suppression of granulomatous reaction.^7^

Decision to discontinue corticosteroids is based on disease activity and end organ damage. When adequate control of the disease is accomplished, they should be tapered off. Optimal duration of treatment is not known. Factors that may facilitate decisions in clinical practice are the appropriate selection of high-risk cases, PET-CT scan, and quality of life parameters. Up to 70% of patients experience sarcoidosis relapse during corticosteroids tapering or after treatment withdrawal. Relapses are treated with the same corticosteroid dose as in acute disease.

Addition of a second anti-inflammatory agent is deemed necessary when disease is severe and continued, when serial relapses occur, when corticosteroids are unable to be tapered below 10 to 15 mg of prednisone, when toxicity is significant, and when disease does not respond to corticosteroids.^26^

### Methotrexate

The most commonly studied second-line agent in sarcoidosis is antimetabolite methotrexate (MTX). MTX has exhibited a higher efficacy rate among second-line options managing disease control and steroids tapering in about 65% to 85% of patients with persistent pulmonary sarcoidosis.^26,27,28,29^ MTX has also shown improvement in radiology and in FVC but only in observational or retrospective studies.^26,27,28,29^ Additionally, MTX has proven effective in neurosarcoidosis, cardiac disease, skin, eye, and disrupted calcium metabolism.^1^ It is given at a total dose of 15 mg weekly, divided in one or two doses. The most common side effects are blood (cytopenias) and liver toxicities, so these parameters should be monitored closely. To prevent bone marrow toxicity, concomitant treatment with folic acid is imperative. Less than 10% of patients have to stop MTX because of toxicity, and relapses after MTX withdrawal may occur.^28^ When initiating MTX, males and females of child-bearing age should be warned to prevent fertility due to fetal toxicity during pregnancy. Based on the existing evidence and acceptable toxicity profile, MTX is the recommended first option for a steroid-sparing second-line agent when treating sarcoidosis.^14,26^

### Azathioprine

Azathioprine (AZA) is a second antimetabolite that has long been used for rheumatic and hematologic diseases and historically has been used in sarcoidosis. Its efficacy is considered less than that of MTX, ranging between 20% to 80%.^29,30^ However, these data come from retrospective or observational studies only. Additionally, AZA has significant side effects, mainly gastrointestinal issues, myelodysplasia, malignancies, and secondary infections, with treatment cessation more frequent than MTX. Recent European Respiratory Society (ERS) guidelines made no recommendation for its use as a steroid-sparing agent in sarcoidosis.^14^

### Leflunomide

Leflunomide is a cytotoxic drug used in rheumatoid arthritis. Small series have shown efficacy of leflunomide at a dose of 10 to 20 mg daily in pulmonary and extrapulmonary sarcoidosis to improve pulmonary physiology, quality of life, extrapulmonary organ response, and as a steroid-sparing agent.^31,32^ Leflunomide is typically used when a need arises for a second agent with a better safety toxicity profile than MTX. However, leflunomide has adverse effects, most significantly interstitial pneumonia and peripheral neuropathy.^33,34^

### Hydroxychloroquine

Chloroquine and hydroxychloroquine (HCQ) are antimalaria drugs used to treat sarcoidosis. Recently, HCQ is used more often due to its lower toxicity profile, although retinopathy remains a significant concern, prompting ophthalmology evaluation upon symptoms.^35^ HCQ has shown efficacy in cutaneous sarcoidosis and hypercalcemia.^36,37^ In these conditions, it is usually given as an adjunct to low-dose corticosteroids or monotherapy. One randomized trial showed some efficacy in pulmonary sarcoidosis and physiology.^38^ Considered less potent than MTX or other second-line agents, HCQ may be considered in refractory cases when drug toxicity is significant and in combination with one or two other drugs.

### Mycophenolate Mofetil

Mycophenolate mofetil (MMF) is a powerful inhibitor of lymphocyte proliferation, used to prevent allograft rejection after transplant. Used in various rheumatic diseases, its role in sarcoidosis remains unclear. Retrospective studies showed that MMF has a steroid-sparing effect in chronic sarcoidosis and neurosarcoidosis but is probably less effective than other second-line agents (mainly MTX), with higher relapse rates and a more favorable toxicity profile.^39,40,41^ Its specific position in the treatment pyramid, likely as a second-line agent, must be further elucidated.

### Infliximab and Other Biologic Agents

Anti-tumor necrosis factor-α (anti-TNF-α) agents are considered effective third-line drugs in sarcoidosis (especially with refractory pulmonary disease and neurosarcoidosis) by blocking TNF-α, a significant cytokine in granulomatous inflammation. In particular, infliximab (and biosimilars) have shown improvement in FVC in two double-blind randomized studies and was granted a conditional recommendation for use in refractory pulmonary sarcoidosis under CS with or without a second-line agent.^42,43^ Infliximab is further proven effective in lupus pernio (efficacy 77%), cutaneoussarcoidosis, and neurosarcoidosis (clinical improvement 77%, imaging improvement 82%).^44,45^ Clinicians must be aware of side effects, ie, infections and tuberculosis.^46,47^ Adalimumab may be an alternative in case of infliximab intolerance, as shown (at least partly) in small open-label studies.^48^ Conversely, neither golimumab nor ustekinumab (anti-IL12/23) showed efficacy in refractory pulmonary sarcoidosis in one randomized trial.^49^

### Other Treatments

Various other drugs have been tested in sarcoidosis. Phosphodiesterase inhibitors, pentoxifylline, roflumilast, and apremilast have shown to reduce disease flares, improve quality of life, and improve cutaneous disease, respectively, in small studies.^50,51,52^ Rituximab is an anti-CD20 B-lymphocytes antibody that has shown partial efficacy in small case series of advanced pulmonary, eye, and neurosarcoidosis, with a favorable tolerance profile, and may be considered a third-line option.^53^ Antimycobacterial treatment (CLEAR regimen) for 4 months failed to demonstrate clinical or physiological improvement in chronic pulmonary sarcoidosis.^54^ Repository corticotropin injection (RCI) was approved in the 1950s for sarcoidosis. Originally considered a steroid analog, RCI may have additional immunological properties and a steroid-sparing effect, although its action is currently limited.^55^ Janus kinase inhibitors are a drug family recently introduced in sarcoidosis treatment. Among them, tofacitinib showed 60% steroid-sparing efficacy in chronic sarcoidosis in a small proof-of-concept study.^56^ An overview of drugs used, dosages, and respective monitoring events, with a focus on CS, is presented in ***Table 2***.

**Table 2:** Drugs used to treat sarcoidosis. qd: every day; bid: twice a day


DRUG	DOSE	SIDE EFFECTS	CARDIAC SARCOIDOSIS

Prednisone	20 mg qd Follow-up 5-10 mg qd	Diabetes Weight gain Osteoporosis Glaucoma Cataract Depression/psychosis	Initiating dose 30 mg qd

Methotrexate	10-15 mg once a week	Nausea, bone marrow suppression, liver toxicity, pulmonary toxicity	Effective in combination with corticosteroids

Leflunomide	10-20 mg qd	Nausea, peripheral neuropathy, interstitial pneumonia	

Azathioprine	50-250 mg qd	Nausea, bone marrow suppression, liver toxicity, malignancies	Less effective than methotrexate

Mycophenolate mofetil	500-1500 mg bid	Diarrhea, monitor blood cell counts	Reasonably effective

Infliximab	3-5 mg/kg initially, 2nd dose after 2 weeks, then every 4-6 weeks	Tuberculosis activation, infections, contraindicated in severe heart failure, demyelinating neurological diseases, active tuberculosis, prior malignancy	Effective, caution for infections, cardiac symptoms^47^

Adalimumab	40 mg every 1-2 weeks	Tuberculosis activation, infections Contraindicated in severe heart failure, demyelinating neurological diseases, active tuberculosis, prior malignancy	Less effective than infliximab, caution for infections, cardiac symptoms^47^

Rituximab	500-1000 mg every 1-6 months	Screen for hepatitis, tuberculosis activation, humoral deficiency	

Hydroxychloroquine	200-400 mg qd	Retinopathy	


## Specific Considerations

### Extrapulmonary Sarcoidosis

Depending on organ involvement, extrapulmonary sarcoidosis may warrant local treatment, systemic treatment escalation, or multimodality interventions.

#### Cutaneous Sarcoidosis

Cutaneous sarcoidosis should initially be treated with local or intralesional corticosteroid injection. Cosmetically important disfiguring lesions (such as lupus pernio) should be treated with oral corticosteroids to achieve control in two-thirds of cases.^57^ Continued disease should be treated with an additional agent. Hydroxychloroquine and methotrexate are currently used and have shown efficacy in small studies.^58^ Randomized studies provide support for the use of infliximab in severe skin disease.^44,59^ Thalidomide, on the other hand, is proven ineffective compared with a placebo in skin sarcoidosis.^60^

#### Ocular Sarcoidosis

Anterior uveitis should be treated locally with steroidal eye drops. Posterior uveitis, however, carries risk for blindness and requires systemic treatment, which includes corticosteroids and/or methotrexate in the majority of cases.^61^ Mycophenolate mofetil and infliximab may be used as well, depending on course and individual toxicities.

#### Calcium Metabolism

Hypercalciuria is treated initially with dietary dairy and restricted sun exposure while hypercalcemia requires systemic treatment. Corticosteroids and/or HCQ are effective in treating hypercalcemia, the latter as sole treatment particularly in cases of unacceptable corticosteroids toxicity.^36^ Osteoporosis should be treated carefully in sarcoidosis, as exogenous vitamin D supplementation may lead to hypercalcemia and is only indicated when 1,25(OH)_2_ vitamin D levels are normal or reduced. When 1,25(OH)_2_ vitamin D is elevated, osteoporosis is treated with bisphosphonates or denosumab.^62^

#### Cardiac Sarcoidosis

Treatment of CS is two-fold. First, based on current guidelines and cardiology consultation, patients at increased risk for sudden cardiac death should be recognized and given an implantable cardioverter defibrillator (ICD), as in all arrhythmiogenic cardiomyopathies. Such patients include those with episode of arrest, left ventricle ejection fraction (LVEF) ≤ 35%, and sustained ventricular tachycardia but also when LVEF is > 35% along with certain physiological or functional features.^63,64,65^ When no indication for ICD exists, performing an electrophysiology study is reasonable. Anti-arrhythmic drugs, pacemaker placement in high A-V blocks, and catheter ablation have been of modest value.^66^

Second, patients with clinically relevant cardiac sarcoidosis should initiate anti-inflammatory treatment with corticosteroids. Studies have shown long-term benefit of early initiation of immunosuppressives in CS. Corticosteroids in particular have shown survival and functional benefit, albeit not in randomized trials; lower doses of prednisone (no more than 30 mg) are not inferior to higher doses and are preferred.^67,68,69,70^ Ongoing studies are underway to evaluate prospectively the impact and dose of corticosteroids in CS (Cardiac Sarcoidosis Multi-center Cohort Study, NCT01477359). Regarding second- and third-line agents, functional benefit is shown with MTX, MMF, infliximab, and adalimumab.^71,72,73,74^ In most of these studies, adding a second agent was superior to treatment with corticosteroids alone. Anti-TNF agents were not associated with heart failure deterioration in these studies of CS patients. Unanswered questions remain as per the duration of treatment, the value of adding a second agent from the beginning or later in the course of the disease as well as the management of clinically asymptomatic (ie, normal rhythm and ejection fraction) cardiac involvement.^75^ FDG-PET seems a promising biomarker to serially evaluate cardiac inflammation and response to treatment.^15^

#### Pulmonary Hypertension

Sarcoidosis-associated pulmonary hypertension (SAPH) is encountered in 5% to 20% of sarcoidosis patients. SAPH along with pulmonary fibrosis constitute an advanced sarcoidosis phenotype, ie, sarcoidosis with a risk of significant loss of organ function or death.^76^ When SAPH is suspected, right heart catheterization is necessary for confirmation and differentiation of multiple SAPH etiologies (ie, left heart failure, precapillary PH, extrinsic compression, and pulmonary veno-occlusive disease). Precapillary SAPH carries a 3-year mortality of 30%. Worse outcomes relate to reduced DLCO < 35%, reduced 6-minute walk distance < 300 m, and preserved FEV1/FVC ratio.^77^ In a small series, epoprostenol and combined ambrisentan with tadalafil showed hemodynamic and functional improvement.^78,79^ As to randomized trials, bosentan improved pulmonary hemodynamics at 4 months of treatment, while riociguat significantly delayed time to clinical worsening and improved exercise capacity at 1 year of treatment.^80,81^

#### Pulmonary Fibrosis

Pulmonary fibrosis extending > 20% of the lungs in high-resolution CT implies advanced disease with high mortality risk.^11,12^ Transplant referral is indicated when sarcoidosis progresses to respiratory failure despite treatment. Transplant has been performed in patients with fibrosis or obstructive or mixed defects with or without pulmonary hypertension. Ensuing 5-year survival rates were 69%, with age and fibrosis being the worst prognostic factors.^82^

Antifibrotic treatment with nintedanib in sarcoidosis with pulmonary fibrosis has shown to ameliorate vital capacity decline, as it did with other progressive fibrosing interstitial lung diseases, in the IN-BUILD trial and may be considered pretransplant.^83^ A study exploring pirfenidone in fibrotic sarcoidosis is currently underway.

#### Neurosarcoidosis

Occurring in 5% to 20% of sarcoidosis, neurosarcoidosis manifests mainly as cranial nerve palsy, parenchymal masses, hydrocephalus, or aseptic meningitis and is associated with a 10-year mortality of 5% to 10%.^84,85^ Although data are derived from retrospective studies and meta-analyses, they support initiation of corticosteroids versus no treatment, with efficacy rates of corticosteroids alone up to 70%.^84,85^ The dose usually administered in clinical practice is prednisone ≥ 0.5 mg/kg daily. A second-line agent will probably be required in continued or relapsing disease, with MTX exhibiting superiority versus MMF, azathioprine, and HCQ (response in 55-70% of cases).^84,86^ Lastly, about a third of patients with neurosarcoidosis may require a third-line agent concomitantly. Infliximab is mostly efficient as shown in two studies and less toxic than cyclophosphamide, but relapses tend to occur after treatment discontinuation.^45,87^

**Figure 1:**
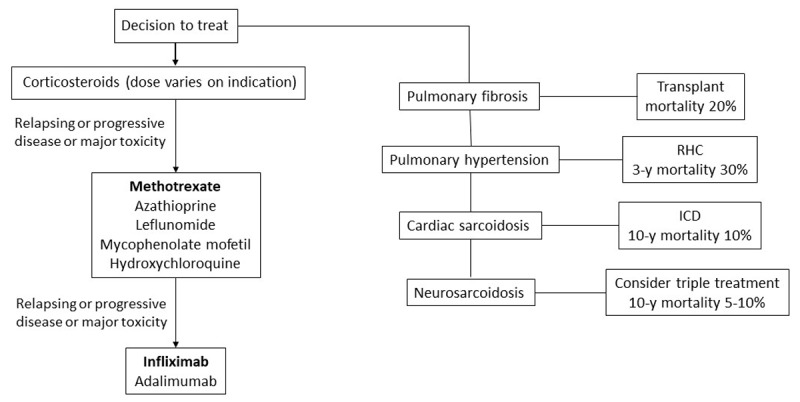
Schema for sarcoidosis treatment. Systemic treatment initiates regularly with corticosteroids, with dose depending on indication. Among second- and third-line treatments, methotrexate and infliximab (in bold) have the most evidence for use. Agent selection depends on indication, comorbidities, and anticipated toxicity. A proportion of these patients develop features of advanced sarcoidosis (right column) with respective mortality risk where additional treatment modalities may be required. RHC: right heart catheterization; ICD: implantable cardioverter defibrillator^14,69,76,81,83,84^

***Figure 1*** depicts lines of treatment with available regimens and, on the right-hand side, specific considerations in advanced disease.

### Fatigue

Fatigue affects ≥ 60% of patients with sarcoidosis, compromising quality of life, yet it is not directly related to disease activity of the granulomas. Fatigue may be multifactorial due to diabetes, thyroid disease, sleep apnea, depression, and neuropathies. Interventions to alleviate fatigue include pulmonary rehabilitation, neurostimulants, and low-dose corticosteroids. Pulmonary rehabilitation has shown in several studies to improve physical activity, exercise capacity, or just the levels of fatigue.^87,88^ Inspiratory muscle training, a component of pulmonary rehabilitation, may add benefit.^89^ Pulmonary rehabilitation should be offered in sarcoidosis, as with all interstitial lung diseases. Dexmethylphenidate hydrochloride and armodafinil improved fatigue in 10 and 15 patients with sarcoidosis, respectively, but their use is restricted by side effects such as insomnia and anxiety.^90,91^ Lastly, in a randomized trial of sarcoidosis subjects without indication for immunosuppressant treatment, 1 mg dexamethasone daily for 12 months improved fatigue parameters along with serum inflammatory markers; however, this intervention needs confirmation.^92^

### Small Fiber Neuropathy

Small fiber neuropathy relates to many diseases, is characterized by debilitating neuropathic symptoms—mainly pain and dysautonomia—and occurs in up to 60% of sarcoidosis patients.^93^ Often under-recognized, diagnosis requires large fiber disease exclusion and is facilitated by skin biopsy and sudomotor testing.^94^ Since there is no established treatment, palliative treatment on a case-by-case basis may include tramadol, anticonvulsants, topiramate, gamma-aminobutyric acid analogues, and intravenous immunoglobulin.^95^

## Conclusion

Treatment of a patient with sarcoidosis should take into account not only physiologic impairment of the organ but also quality of life and patient-reported outcomes. Corticosteroids, the first-line treatment for acute and chronic disease, should be employed at the lowest affordable dose. To allow this, steroid-sparing agents, in particular methotrexate, are useful and effective. Infliximab, among anti-TNF agents, is effective in refractory pulmonary, cutaneous, and neurosarcoidosis. Patients with advanced disease, pulmonary fibrosis, pulmonary hypertension, cardiac sarcoidosis, and neurosarcoidosis should be thoroughly identified and treated accordingly. Fatigue and small fiber neuropathy impose significant burdens to patients, and efforts should be made to relieve them.

## Key Points

- Treatment goals in sarcoidosis are preventing permanent end-organ dysfunction and preserving quality of life.
- Corticosteroids remain the first-line treatment, although with significant cumulative side effects.
- Second- and third-line agents may be used in refractory or relapsing disease or when corticosteroids cause unacceptable toxicity; methotrexate and infliximab have shown important efficacy with acceptable safety profile in cardiac sarcoidosis.
- Advanced sarcoidosis refers to high mortality disease manifestations (pulmonary fibrosis, pulmonary hypertension, cardiac sarcoidosis, and neurosarcoidosis) and necessitates treatment.
- Cardiac sarcoidosis requires immunosuppressive treatment and recognition of patients at risk for sustained arrythmias, which have to be managed thoroughly.
- Long-term maintenance therapy remains controversial.

## References

[1] Hunninghake GW, Costabel U, Ando M, et al. ATS/ERS/WASOG statement on sarcoidosis. American Thoracic Society/European Respiratory Society/World Association of Sarcoidosis and other G ranulomatous Disorders. Sarcoidosis Vasc Diffuse Lung Dis. 1999 Sep;16(2):149-73. 10560120

[2] Crouser ED, Maier LA, Wilson KC, et al. Diagnosis and Detection of Sarcoidosis. An Official American Thoracic Society Clinical Practice Guideline. Am J Respir Crit Care Med. 2020 Apr 15;201(8):e26-e51. doi: 10.1164/rccm.202002-0251ST 32293205 PMC7159433

[3] Birnie D, Ha AC, Gula LJ, Chakrabarti S, Beanlands RS, Nery P. Cardiac Sarcoidosis. Clin Chest Med. 2015 Dec;36(4):657-68. doi: 10.1016/j.ccm.2015.08.008 26593140

[4] Kandolin R, Lehtonen J, Airaksinen J, et al. Cardiac sarcoidosis: epidemiology, characteristics, and outcome over 25 years in a nationwide study. Circulation. 2015 Feb 17;131(7):624-32. doi: 10.1161/CIRCULATIONAHA.114.011522 25527698

[5] Baughman RP, Nagai S, Balter M, et al. Defining the clinical outcome status (COS) in sarcoidosis: results of WASOG Task Force. Sarcoidosis Vasc Diffuse Lung Dis. 2011 Jul;28(1):56-64. 21796892

[6] Thillai M, Atkins CP, Crawshaw A, et al. BTS Clinical Statement on pulmonary sarcoidosis. Thorax. 2021 Jan;76(1):4-20. doi: 10.1136/thoraxjnl-2019-214348 33268456

[7] Judson MA. The treatment of pulmonary sarcoidosis. Respir Med. 2012 Oct;106(10):1351-61. doi: 10.1016/j.rmed.2012.01.013 22495110

[8] Terasaki F, Azuma A, Anzai T, et al; Japanese Circulation Society Joint Working Group. JCS 2016 Guideline on Diagnosis and Treatment of Cardiac Sarcoidosis- Digest Version. Circ J. 2019 Oct 25;83(11):2329-2388. doi: 10.1253/circj.CJ-19-0508 31597819

[9] Vita T, Okada DR, Veillet-Chowdhury M, et al. Complementary Value of Cardiac Magnetic Resonance Imaging and Positron Emission Tomography/Computed Tomography in the Assessment of Cardiac Sarcoidosis. Circ Cardiovasc Imaging. 2018 Jan;11(1):e007030. doi: 10.1161/CIRCIMAGING.117.007030 29335272 PMC6381829

[10] Kawai H, Sarai M, Kato Y, et al. Diagnosis of isolated cardiac sarcoidosis based on new guidelines. ESC Heart Fail. 2020 Oct;7(5):2662-2671. doi: 10.1002/ehf2.12853 32578957 PMC7524076

[11] Kirkil G, Lower EE, Baughman RP. Predictors of Mortality in Pulmonary Sarcoidosis. Chest. 2018 Jan;153(1):105-113. doi: 10.1016/j.chest.2017.07.008 28728933

[12] Kouranos V, Ward S, Kokosi MA, et al. Mixed Ventilatory Defects in Pulmonary Sarcoidosis: Prevalence and Clinical Features. Chest. 2020 Nov;158(5):2007-2014. doi: 10.1016/j.chest.2020.04.074 32534908

[13] Birnie DH, Sauer WH, Bogun F, et al. HRS expert consensus statement on the diagnosis and management of arrhythmias associated with cardiac sarcoidosis. Heart Rhythm. 2014 Jul;11(7):1305-23. doi: 10.1016/j.hrthm.2014.03.043 24819193

[14] Baughman RP, Valeyre D, Korsten P, et al. ERS clinical practice guidelines on treatment of sarcoidosis. Eur Respir J. 2021 Dec 16;58(6):2004079. doi: 10.1183/13993003.04079-2020 34140301

[15] Coulden RA, Sonnex EP, Abele JT, Crean AM. Utility of FDG PET and Cardiac MRI in Diagnosis and Monitoring of Immunosuppressive Treatment in Cardiac Sarcoidosis. Radiol Cardiothorac Imaging. 2020 Aug 27;2(4):e190140. doi: 10.1148/ryct.2020190140 33778595 PMC7977729

[16] Paramothayan NS, Lasserson TJ, Jones PW. Corticosteroids for pulmonary sarcoidosis. Cochrane Database Syst Rev. 2005 Apr 18;2005(2):CD001114. doi: 10.1002/14651858.CD001114.pub2 PMC6464973 15846612

[17] James DG, Carstairs LS, Trowell J, Sharma OP. Treatment of sarcoidosis. Report of a controlled therapeutic trial. Lancet. 1967 Sep 9;2(7515):526-8. doi: 10.1016/s0140-6736(67)90493-x 4166889

[18] Zaki MH, Lyons HA, Leilop L, Huang CT. Corticosteroid therapy in sarcoidosis. A five-year, controlled follow-up study. N Y State J Med. 1987 Sep;87(9):496-9. 3478618

[19] Pietinalho A, Tukiainen P, Haahtela T, Persson T, Selroos O; Finnish Pulmonary Sarcoidosis Study Group. Early treatment of stage II sarcoidosis improves 5-year pulmonary function. Chest. 2002 Jan;121(1):24-31. doi: 10.1378/chest.121.1.24 11796428

[20] Gottlieb JE, Israel HL, Steiner RM, Triolo J, Patrick H. Outcome in sarcoidosis. The relationship of relapse to corticosteroid therapy. Chest. 1997 Mar;111(3):623-31. doi: 10.1378/chest.111.3.623 9118698

[21] Broos CE, Poell LHC, Looman CWN, et al. No evidence found for an association between prednisone dose and FVC change in newly-treated pulmonary sarcoidosis. Respir Med. 2018 May;138S:S31-S37. doi: 10.1016/j.rmed.2017.10.022 29137908

[22] Baughman RP, Iannuzzi MC, Lower EE, et al. Use of fluticasone in acute symptomatic pulmonary sarcoidosis. Sarcoidosis Vasc Diffuse Lung Dis. 2002 Oct;19(3):198-204. 12405489

[23] McKinzie BP, Bullington WM, Mazur JE, Judson MA. Efficacy of short-course, low-dose corticosteroid therapy for acute pulmonary sarcoidosis exacerbations. Am J Med Sci. 2010 Jan;339(1):1-4. doi: 10.1097/MAJ.0b013e3181b97635 19996733

[24] Panselinas E, Judson MA. Acute pulmonary exacerbations of sarcoidosis. Chest. 2012 Oct;142(4):827-836. doi: 10.1378/chest.12-1060 23032450

[25] Khan NA, Donatelli CV, Tonelli AR, et al. Toxicity risk from glucocorticoids in sarcoidosis patients. Respir Med. 2017 Nov;132:9-14. doi: 10.1016/j.rmed.2017.09.003 29229111

[26] Baughman RP, Scholand MB, Rahaghi FF. Clinical phenotyping: role in treatment decisions in sarcoidosis. Eur Respir Rev. 2020 Mar 20;29(155):190145. doi: 10.1183/16000617.0145-2019 32198217 PMC9488542

[27] Cremers JP, Drent M, Bast A, et al. Multinational evidence-based World Association of Sarcoidosis and Other Granulomatous Disorders recommendations for the use of methotrexate in sarcoidosis: integrating systematic literature research and expert opinion of sarcoidologists worldwide. Curr Opin Pulm Med. 2013 Sep 1;19(5):545-561. doi: 10.1097/MCP.0b013e3283642a7a 23880702

[28] Fang C, Zhang Q, Wang N, Jing X, Xu Z. Effectiveness and tolerability of methotrexate in pulmonary sarcoidosis: A single center real-world study. Sarcoidosis Vasc Diffuse Lung Dis. 2019;36(3):217-227. doi: 10.36141/svdld.v36i3.8449 32476957 PMC7247084

[29] Vorselaars ADM, Wuyts WA, Vorselaars VMM, et al. Methotrexate vs azathioprine in second-line therapy of sarcoidosis. Chest. 2013 Sep;144(3):805-812. doi: 10.1378/chest.12-1728 23538719

[30] Müller-Quernheim J, Kienast K, Held M, Pfeifer S, Costabel U. Treatment of chronic sarcoidosis with an azathioprine/prednisolone regimen. Eur Respir J. 1999 Nov;14(5):1117-22. doi: 10.1183/09031936.99.14511179 10596700

[31] Sahoo DH, Bandyopadhyay D, Xu M, et al. Effectiveness and safety of leflunomide for pulmonary and extrapulmonary sarcoidosis. Eur Respir J. 2011 Nov;38(5):1145-50. doi: 10.1183/09031936.00195010 21565914

[32] Baughman RP, Lower EE. Leflunomide for chronic sarcoidosis. Sarcoidosis Vasc Diffuse Lung Dis. 2004 Mar;21(1):43-8. doi: 10.1007/s11083-004-5178-y 15127974

[33] Bonnel RA, Graham DJ. Peripheral neuropathy in patients treated with leflunomide. Clin Pharmacol Ther. 2004 Jun;75(6):580-5. doi: 10.1016/j.clpt.2004.01.016 15179412

[34] Raj R, Nugent K. Leflunomide-induced interstitial lung disease (a systematic review). Sarcoidosis Vasc Diffuse Lung Dis. 2013 Nov 22;30(3):167-76. 24284289

[35] Melles RB, Marmor MF. The Prevalence of Hydroxychloroquine Retinopathy and Toxic Dosing, and the Role of the Ophthalmologist in Reducing Both. Am J Ophthalmol. 2016 Oct;170:240. doi: 10.1016/j.ajo.2016.06.045 27544039

[36] Baughman RP, Lower EE. Evidence-based therapy for cutaneous sarcoidosis. Clin Dermatol. May-Jun 2007;25(3):334-40. doi: 10.1016/j.clindermatol.2007.03.011 17560311

[37] Adams JS, Diz MM, Sharma OP. Effective reduction in the serum 1,25-dihydroxyvitamin D and calcium concentration in sarcoidosis-associated hypercalcemia with short-course chloroquine therapy. Ann Intern Med. 1989 Sep 1;111(5):437-8. doi: 10.7326/0003-4819-111-5-437 2764407

[38] Baltzan M, Mehta S, Kirkham TH, Cosio MG. Randomized trial of prolonged chloroquine therapy in advanced pulmonary sarcoidosis. Am J Respir Crit Care Med. 1999 Jul;160(1):192-7. doi: 10.1164/ajrccm.160.1.9809024 10390399

[39] Hamzeh N, Voelker A, Forssén A, et al. Efficacy of mycophenolate mofetil in sarcoidosis. Respir Med. 2014 Nov;108(11):1663-9. doi: 10.1016/j.rmed.2014.09.013 25301291 PMC4254196

[40] Moravan M, Segal BM. Treatment of CNS sarcoidosis with infliximab and mycophenolate mofetil. Neurology. 2009 Jan 27;72(4):337-40. doi: 10.1212/01.wnl.0000341278.26993.22 19171830 PMC2677503

[41] Bitoun S, Bouvry D, Borie R, et al. Treatment of neurosarcoidosis: A comparative study of methotrexate and mycophenolate mofetil. Neurology. 2016 Dec 13;87(24):2517-2521. doi: 10.1212/WNL.0000000000003431 27856779

[42] Baughman RP, Drent M, Kavuru M, et al.; Sarcoidosis Investigators. Infliximab therapy in patients with chronic sarcoidosis and pulmonary involvement. Am J Respir Crit Care Med. 2006 Oct 1;174(7):795-802. doi: 10.1164/rccm.200603-402OC 16840744

[43] Rossman MD, Newman LS, Baughman RP, et al. A double-blinded, randomized, placebo-controlled trial of infliximab in subjects with active pulmonary sarcoidosis. Sarcoidosis Vasc Diffuse Lung Dis. 2006 Oct;23(3):201-8. 18038919

[44] Baughman RP, Judson MA, Lower EE, et al.; Sarcoidosis Investigators. Infliximab for chronic cutaneous sarcoidosis: a subset analysis from a double-blind randomized clinical trial. Sarcoidosis Vasc Diffuse Lung Dis. 2016 Jan 15;32(4):289-95. 26847095

[45] Gelfand JM, Bradshaw MJ, Stern BJ, et al. Infliximab for the treatment of CNS sarcoidosis: A multi-institutional series. Neurology. 2017 Nov 14;89(20):2092-2100. doi: 10.1212/WNL.0000000000004644 29030454 PMC5711506

[46] Keane J, Gershon S, Wise RP, et al. Tuberculosis associated with infliximab, a tumor necrosis factor alpha-neutralizing agent. N Engl J Med. 2001 Oct 11;345(15):1098-104. doi: 10.1056/NEJMoa011110 11596589

[47] Gilotra NA, Wand AL, Pillarisetty A, et al. Clinical and Imaging Response to Tumor Necrosis Factor Alpha Inhibitors in Treatment of Cardiac Sarcoidosis: A Multicenter Experience. J Card Fail. 2021 Jan;27(1):83-91. doi: 10.1016/j.cardfail.2020.08.013 32889044 PMC8350936

[48] Minnis PA, Poland M, Keane MP, Donnelly SC. Adalimumab for refractory pulmonary sarcoidosis. Ir J Med Sci. 2016 Nov;185(4):969-971. doi: 10.1007/s11845-015-1363-9 26428728

[49] Judson MA, Baughman RP, Costabel U, et al. Safety and efficacy of ustekinumab or golimumab in patients with chronic sarcoidosis. Eur Respir J. 2014 Nov;44(5):1296-307. doi: 10.1183/09031936.00000914 25034562

[50] Park MK, Fontana Jr, Babaali H, et al. Steroid-sparing effects of pentoxifylline in pulmonary sarcoidosis. Sarcoidosis Vasc Diffuse Lung Dis. 2009 Jul;26(2):121-31. 20560292 PMC2946799

[51] Baughman RP, Judson MA, Culver DA, et al. Roflumilast (Daliresp®) to reduce acute pulmonary events in fibrotic sarcoidosis: a multi-center, double blind, placebo controlled, randomized clinical trial. Sarcoidosis Vasc Diffuse Lung Dis. 2021;38(3):e2021035. doi: 10.36141/svdld.v38i3.11684 34744427 PMC8552567

[52] Baughman RP, Judson MA, Ingledue R, Craft NL, Lower EE. Efficacy and safety of apremilast in chronic cutaneous sarcoidosis. Arch Dermatol. 2012 Feb;148(2):262-4. doi: 10.1001/archdermatol.2011.301 22004880

[53] Sweiss NJ, Lower EE, Mirsaeidi M, et al. Rituximab in the treatment of refractory pulmonary sarcoidosis. Eur Respir J. 2014 May;43(5):1525-8. doi: 10.1183/09031936.00224513 24488568 PMC4167390

[54] Drake WP, Culver DA, Baughman RP, et al. Phase II Investigation of the Efficacy of Antimycobacterial Therapy in Chronic Pulmonary Sarcoidosis. Chest. 2021 May;159(5):1902-1912. doi: 10.1016/j.chest.2020.12.027 33387486 PMC8129732

[55] Baughman RP, Sweiss N, Keijsers R, et al. Repository corticotropin for Chronic Pulmonary Sarcoidosis. Lung. 2017 Jun;195(3):313-322. doi: 10.1007/s00408-017-9994-4 28353116 PMC5437201

[56] Friedman MA, Le B, Stevens J, et al. Tofacitinib as a Steroid-Sparing Therapy in Pulmonary Sarcoidosis, an Open-Label Prospective Proof-of-Concept Study. Lung. 2021 Apr;199(2):147-153. doi: 10.1007/s00408-021-00436-8 33825964 PMC8092019

[57] Ungprasert P, Wetter DA, Crowson CS, Matteson EL. Epidemiology of cutaneous sarcoidosis, 1976-2013: a population-based study from Olmsted County, Minnesota. J Eur Acad Dermatol Venereol. 2016 Oct;30(10):1799-1804. doi: 10.1111/jdv.13760 27324138 PMC5071110

[58] Jones E, Callen JP. Hydroxychloroquine is effective therapy for control of cutaneous sarcoidal granulomas. J Am Acad Dermatol. 1990 Sep;23(3 Pt 1):487-9. doi: 10.1016/0190-9622(90)70246-e 2212149

[59] Judson MA, Baughman RP, Costabel U, et al.; Centocor T48 Sarcoidosis Investigators. Efficacy of infliximab in extrapulmonary sarcoidosis: results from a randomised trial. Eur Respir J. 2008 Jun;31(6):1189-96. doi: 10.1183/09031936.00051907 18256069

[60] Droitcourt C, Rybojad M, Porcher R, et al. A randomized, investigator-masked, double-blind, placebo-controlled trial on thalidomide in severe cutaneous sarcoidosis. Chest. 2014 Oct;146(4):1046-1054. doi: 10.1378/chest.14-0015 24945194

[61] Baughman RP, Lower EE, Ingledue R, Kaufman AH. Management of ocular sarcoidosis. Sarcoidosis Vasc Diffuse Lung Dis. 2012 Mar;29(1):26-33. PMID: 23311120. 23311120

[62] Baughman RP, Papanikolaou I. Current concepts regarding calcium metabolism and bone health in sarcoidosis. Curr Opin Pulm Med. 2017 Sep;23(5):476-481. doi: 10.1097/MCP.0000000000000400 28598871

[63] Kazmirczak F, Chen KA, Adabag S, et al. Assessment of the 2017 AHA/ACC/HRS Guideline Recommendations for Implantable Cardioverter-Defibrillator Implantation in Cardiac Sarcoidosis. Circ Arrhythm Electrophysiol. 2019 Sep;12(9):e007488. doi: 10.1161/CIRCEP.119.007488 31431050 PMC6709696

[64] Al-Khatib SM, Stevenson WG, Ackerman MJ, et al. 2017 AHA/ACC/HRS Guideline for Management of Patients With Ventricular Arrhythmias and the Prevention of Sudden Cardiac Death: A Report of the American College of Cardiology/American Heart Association Task Force on Clinical Practice Guidelines and the Heart Rhythm Society. J Am Coll Cardiol. 2018 Oct 2;72(14):e91-e220. doi: 10.1016/j.jacc.2017.10.054 29097296

[65] Towbin JA, McKenna WJ, et al. 2019 HRS expert consensus statement on evaluation, risk stratification, and management of arrhythmogenic cardiomyopathy. Heart Rhythm. 2019 Nov;16(11):e301-e372. doi: 10.1016/j.hrthm.2019.05.007 31078652

[66] Jefic D, Joel B, Good E, et al. Role of radiofrequency catheter ablation of ventricular tachycardia in cardiac sarcoidosis: report from a multicenter registry. Heart Rhythm. 2009 Feb;6(2):189-95. doi: 10.1016/j.hrthm.2008.10.039 19187909

[67] Padala SK, Peaslee S, Sidhu MS, Steckman DA, Judson MA. Impact of early initiation of corticosteroid therapy on cardiac function and rhythm in patients with cardiac sarcoidosis. Int J Cardiol. 2017 Jan 15;227:565-570. doi: 10.1016/j.ijcard.2016.10.101 27836297

[68] Yazaki Y, Isobe M, Hiroe M, et al; Central Japan Heart Study Group. Prognostic determinants of long-term survival in Japanese patients with cardiac sarcoidosis treated with prednisone. Am J Cardiol. 2001 Nov 1;88(9):1006-10. doi: 10.1016/s0002-9149(01)01978-6 11703997

[69] Chapelon-Abric C, de Zuttere D, Duhaut P, et al. Cardiac sarcoidosis: a retrospective study of 41 cases. Medicine (Baltimore). 2004 Nov;83(6):315-334. doi: 10.1097/01.md.0000145367 15525844

[70] Zhou Y, Lower EE, Li HP, Costea A, Attari M, Baughman RP. Cardiac Sarcoidosis: The Impact of Age and Implanted Devices on Survival. Chest. 2017 Jan;151(1):139-148. doi: 10.1016/j.chest.2016.08.1457 27614001

[71] Nagai S, Yokomatsu T, Tanizawa K, et al. Treatment with methotrexate and low-dose corticosteroids in sarcoidosis patients with cardiac lesions. Intern Med. 2014;53(23):2761. doi: 10.2169/internalmedicine.53.3120. 25447669

[72] Rosenthal DG, Parwani P, Murray TO, et al. Long-Term Corticosteroid-Sparing Immunosuppression for Cardiac Sarcoidosis. J Am Heart Assoc. 2019 Sep 17;8(18):e010952. doi: 10.1161/JAHA.118.010952 31538835 PMC6818011

[73] Griffin JM, Chasler J, Wand AL, et al. Management of Cardiac Sarcoidosis Using Mycophenolate Mofetil as a Steroid-Sparing Agent. J Card Fail. 2021 Dec;27(12):1348-1358. doi: 10.1016/j.cardfail.2021.06.010 34166800

[74] Baker MC, Sheth K, Witteles R, Genovese MC, Shoor S, Simard JF. TNF-alpha inhibition for the treatment of cardiac sarcoidosis. Semin Arthritis Rheum. 2020 Jun;50(3):546-552. doi: 10.1016/j.semarthrit.2019.11.004 31806154 PMC7225041

[75] Ballul T, Borie R, Crestani B, et al. Treatment of cardiac sarcoidosis: A comparative study of steroids and steroids plus immunosuppressive drugs. Int J Cardiol. 2019 Feb 1;276:208-211. doi: 10.1016/j.ijcard.2018.11.131 30527995

[76] Gupta R, Judson MA, Baughman RP. Management of Advanced Pulmonary Sarcoidosis. Am J Respir Crit Care Med. 2021 Nov 23. doi: 10.1164/rccm.202106-1366CI. Online ahead of print. 34813386

[77] Shlobin OA, Kouranos V, Barnett SD, et al. Physiological predictors of survival in patients with sarcoidosis-associated pulmonary hypertension: results from an international registry. Eur Respir J. 2020 May 14;55(5):1901747. doi: 10.1183/13993003.01747-2019 32139456

[78] Abston E, Moll M, Hon S, Govender P, Berman J, Farber H. Long-term outcomes of epoprostenol therapy in sarcoid associated pulmonary hypertension. Sarcoidosis Vasc Diffuse Lung Dis. 2020;37(2):184-191. doi: 10.36141/svdld.v37i2.9150 33093782 PMC7569545

[79] Abston E, Hon S, Lawrence R, Berman J, Govender P, Farber HW. Treatment of newly diagnosed sarcoid-associated pulmonary hypertension with ambrisentan and tadalafil combination therapy. Sarcoidosis Vasc Diffuse Lung Dis. 2020;37(2):234-238. doi: 10.36141/svdld.v37i2.9343 33093789 PMC7569547

[80] Baughman RP, Culver DA, Cordova FC, et al. Bosentan for sarcoidosis-associated pulmonary hypertension: a double-blind placebo controlled randomized trial. Chest. 2014 Apr;145(4):810-817. doi: 10.1378/chest.13-1766 24177203

[81] Baughman RP, Shlobin OA, Gupta R, et al. Riociguat for Sarcoidosis-Associated Pulmonary Hypertension: Results of a 1-Year Double-Blind, Placebo-Controlled Trial. Chest. 2021 Aug 4:S0012-3692(21)03613-8. doi: 10.1016/j.chest.2021.07.2162 PMC9005858 34363816

[82] Le Pavec J, Valeyre D, Gazengel P, et al. Lung transplantation for sarcoidosis: outcome and prognostic factors. Eur Respir J. 2021 Aug 5;58(2):2003358. doi: 10.1183/13993003.03358-2020 33479107

[83] Flaherty KR, Wells AU, Cottin V, et al.; INBUILD trial investigators. Nintedanib in progressive interstitial lung diseases: data from the whole INBUILD trial. Eur Respir J. 2021 Sep 2:2004538. doi: 10.1183/13993003.04538-2020. Online ahead of print. PMC8927709 34475231

[84] Fritz D, van de Beek D, Brouwer MC. Clinical features, treatment and outcome in neurosarcoidosis: systematic review and meta-analysis. BMC Neurol. 2016 Nov 15;16(1):220. doi: 10.1186/s12883-016-0741-x 27846819 PMC5109654

[85] Joubert B, Chapelon-Abric C, Biard L, et al. Association of Prognostic Factors and Immunosuppressive Treatment With Long-term Outcomes in Neurosarcoidosis. JAMA Neurol. 2017 Nov 1;74(11):1336-1344. doi: 10.1001/jamaneurol.2017.2492 29052709 PMC5710577

[86] Lower EE, Broderick JP, Brott TG, Baughman RP. Diagnosis and management of neurological sarcoidosis. Arch Intern Med. 1997 Sep 8;157(16):1864-8. PMID: 9290546. 9290546

[87] Cohen Aubart F, Bouvry D, Galanaud D, et al. Long-term outcomes of refractory neurosarcoidosis treated with infliximab. J Neurol. 2017 May;264(5):891-897. doi: 10.1007/s00415-017-8444-9 28260120

[88] Naz I, Ozalevli S, Ozkan S, Sahin H. Efficacy of a Structured Exercise Program for Improving Functional Capacity and Quality of Life in Patients With Stage 3 and 4 Sarcoidosis: A RANDOMIZED CONTROLLED TRIAL. J Cardiopulm Rehabil Prev. 2018 Mar;38(2):124-130. doi: 10.1097/HCR.0000000000000307 29401114

[89] Wallaert B, Kyheng M, Labreuche J, Stelianides S, Wemeau L, Grosbois JM. Long-term effects of pulmonary rehabilitation on daily life physical activity of patients with stage IV sarcoidosis: A randomized controlled trial. Respir Med Res. 2020 Mar;77:1-7. doi: 10.1016/j.resmer.2019.10.003 31855785

[90] Karadallı MN, Boşnak-Güçlü M, Camcıoğlu B, Kokturk N, Türktaş H. Effects of Inspiratory Muscle Training in Subjects With Sarcoidosis: A Randomized Controlled Clinical Trial. Respir Care. 2016 Apr;61(4):483-94. doi: 10.4187/respcare.04312 26715771

[91] Lower EE, Harman S, Baughman RP. Double-blind, randomized trial of dexmethylphenidate hydrochloride for the treatment of sarcoidosis-associated fatigue. Chest. 2008 May;133(5):1189-95. doi: 10.1378/chest.07-2952 18263672

[92] Lower EE, Malhotra A, Surdulescu V, Baughman RP. Armodafinil for sarcoidosis-associated fatigue: a double-blind, placebo-controlled, crossover trial. J Pain Symptom Manage. 2013 Feb;45(2):159-69. doi: 10.1016/j.jpainsymman.2012.02.016 22917711 PMC3678278

[93] Vis R, van de Garde EMW, Meek B, Korenromp IHE, Grutters JC. Randomised, placebo-controlled trial of dexamethasone for quality of life in pulmonary sarcoidosis. Respir Med. 2020 Apr-May;165:105936. doi: 10.1016/j.rmed.2020.105936 32308204

[94] Tavee J, Culver D. Sarcoidosis and small-fiber neuropathy. Curr Pain Headache Rep. 2011 Jun;15(3):201-6. doi: 10.1007/s11916-011-0180-8 21298560

[95] Tavee JO, Karwa K, Ahmed Z, Thompson N, Parambil J, Culver DA. Sarcoidosis-associated small fiber neuropathy in a large cohort: Clinical aspects and response to IVIG and anti-TNF alpha treatment. Respir Med. 2017 May;126:135-138. doi: 10.1016/j.rmed.2017.03.011 28318820

